# The predictive value of pre- and post-induction chemotherapy plasma EBV DNA level and tumor volume for the radiosensitivity of locally advanced nasopharyngeal carcinoma

**DOI:** 10.17179/excli2017-752

**Published:** 2017-11-28

**Authors:** Yang Song, He Xiao, Zhenzhou Yang, Mingying Geng, Jungang Ma, Yujiang Ren, Yun Liu, Ge Wang

**Affiliations:** 1 Department of Oncology, Third Affiliated Hospital of Third Military Medical University, Chongqing, China

**Keywords:** nasopharyngeal carcinoma, Epstein-Barr virus, induction chemotherapy, intensity-modulated radiation therapy, radiosensitivity, logistic regression

## Abstract

This study was dedicated to investigate the predictive value of pre- and post-induction chemotherapy plasma EBV (Epstein-Barr Virus) DNA level and tumor volume for the radiosensitivity of locally advanced NPC. 129 previously untreated locally advanced NPC patients were enrolled. Plasma EBV-DNA copy number and tumor volume was detected before and after induction chemotherapy. The tumor volume was also measured after radiotherapy. Among 129 patients, 98 were positive for EBV DNA. The residual gross target volume of the primary tumor (GTVnx) and GTVnd after radiotherapy was positively correlated with post-induction chemotherapy EBV copy number (rho=0.357, *P*<0.001; rho=0.356, *P*<0.001, respectively). Univariate logistic regression analyses showed that the AUC of ROC curves of post-induction chemotherapy tumor volume, tumor regression rate before and after induction chemotherapy, post-induction EBV copy number, EBV decrease rate for predicting no residual nasopharyngeal tumor were 0.859, 0.782, 0.678 and 0.657, respectively. Multivariate logistic analyses showed that T stage, post-induction chemotherapy EBV copy number and tumor volume were independent predictors for no residual nasopharyngeal tumor after radiotherapy. The changes in plasma EBV DNA and tumor volume during treatment could be used to predict the sensitivity of locally advanced NPC patients in response to intensity-modulated radiation therapy (IMRT).

## Introduction

Nasopharyngeal carcinoma (NPC) is a malignant tumor with ethnic and regional aggregation, especially in Southeast Asia and southern China (Lee et al., 2012[5]; Zhang et al., 2013[14]). Due to its insidious onset in early stages, NPC has usually been in its locally advanced stage when diagnosed. According to the NCCN guidelines, platinum-based concurrent chemoradiotherapy with or without induction chemotherapy is the preferred treatment for locally advanced NPC. Moreover, the guideline recommends a close monitor of the level of Epstein-Barr virus (EBV) DNA during both treatment and follow-up, as numerous studies have identified EBV DNA levels as a marker of tumor burden, tumor recurrence monitoring and prognosis (Song and Yang, 2013[10]; Lin et al., 2004[6][7]; Twu et al., 2007[12]). Nevertheless, it remains a question whether concurrent chemotherapy can provide higher treatment efficacy to all locally advanced NPC patients who undergo intensity-modulated radiation therapy (IMRT). Some studies have suggested that individualized treatment plan should be formulated based on the specific risk factors of each patient (Lin et al., 2004[6]), which requires a rapid, accurate and sensitive predictor for individual radiosensitivity of the patient. Although tumor burden and EBV DNA level during treatment are the most reliable indicators for the evaluation of short-and long-term efficacy, the predictive values of EBV DNA level and tumor volume before and after induction chemotherapy for tumor radiosensitivity has not been investigated. In this retrospective study, we measured the EBV DNA level and tumor volume in 129 locally advanced NPC patients who received 2 cycles of induction chemotherapy and concurrent chemoradiotherapy, and analyzed the predictive factors for tumor radiosensitivity through a logistic regression model. The current study shall provide a theoretical and practical basis for the development of individualized treatment of NPC. 

## Materials and Methods

### Patient information

This study included 129 previously untreated locally advanced NPC patients who were diagnosed and given induction chemotherapy and concurrent chemoradiotherapy at the Department of Oncology-Pathology in our hospital. Before the treatment, all patients underwent physical examination, electronic nasopharyngoscopy, nasopharyngeal contrast-enhanced MRI, neck chest and abdomen contrast-enhanced CT scan, whole body bone scan, blood biochemical examination, EBV-DNA copy number, and thyroid function test. The patient inclusion criteria were as follows: (1) pathologically confirmed NPC; (2) locally advanced stage III/IVA NPC without distant organ metastasis (2009 UICC staging system); (3) PS score between 0 and 1; (4) completion of 2 cycles of TP or TPF-based induction chemotherapy, followed by concurrent IMRT and chemotherapy; and (5) availability of complete clinical data. Written informed consent was obtained from all participants. This study was approved by the Medical Ethics Committee of Daping Hospital. Table 1(Tab. 1) summarizes the patient information.

### Treatment

#### Chemotherapy 

Patients were given 2 cycles of TP or TPF-based induction chemotherapy. The TP regimen consisted of docetaxel 75 mg/m^2^ or paclitaxel 135 mg/m^2^ on d1 + nedaplatin 80 mg/m^2^ on d1-3. The TPF regimen involved 120 h continuous intravenous infusion of docetaxel 75 mg/m^2^ or paclitaxel 135 mg/m^2^ on d1 + nedaplatin 80 mg/m^2^ on d1-3 + fluorouracil 450-550 mg/m^2^ on d1-5. Each cycle was completed in 4 weeks and a patient was given 2 cycles. Patients were then given concurrent radiotherapy and 2 cycles of TP chemotherapy as described above.

#### Radiotherapy 

All patients were given IMRT with a 6-MV linear accelerator (Elekta, Sweden). The treatment target was outlined based on pre-treatment MRI results, and the ICRU report 50 and 62. The single dose for the gross target volume of the primary tumor (GTVnx) was 2.12-2.2 Gy, resulting in a total dose of 70-72.6 Gy. The single dose for the gross target volume of the metastatic lymph nodes (GTVnd) was 2.00 Gy, and the total dose was 66 Gy. The maximum dose for critical organs was determined according to the RTOG-0615 protocol. 

### Measurement of tumor volume

Once the tumor was outlined, the GTVnx and GTVnd were automatically calculated with an Elekta TPS treatment planning system (TPS). The gross tumour volume (GTV) was then calculated accordingly.

### Detection of plasma EBV DNA level

Fasting blood (3 ml) of patients was collected using an EDTA vacuum blood collection tube before and after the induction chemotherapy. The plasma was isolated by centrifugation at 1500 g for 10 min and stored at -80 °C until use. Free DNA was extracted from the plasma using a QIAamp DNA blood kit. Plasma EBV DNA copy number was determined by TaqMan real-time PCR assay using a BioRad CFX 96TM real-time PCR instrument (Bio-Rad Laboratories Inc., America) (Lo et al., 1999[8]). The standard curve was made using the human Burkitt's lymphoma cell line Namalwa (Cobioer Biosciences Co., Ltd Nanjing, China) as a standard. Namalwa genomic DNA was extracted and quantified using a NanoDrop2000 (Thermo Fisher Scientific Inc., America). The weight of each diploid cell genome was set as 6.6 pg to calculate the copy number of standard. Forward primer: 5'-CCCAACACTCCACCACACC-3', reverse primer: 5'-TCTTAGGA GCTGTCCGAGGG-3', fluorescent probe: 5'-(FAM)CACACACTACACACACCCACCCGTCTC(TAMR A)-3'. Both primers and Taqman fluorescent probes were synthesized by Daan Gene Co., Ltd. at the Sun Yat-sen University (Guangzhou, China). The reaction mixture was prepared (50 μl) and thermal reaction was as follows: 95 °C denaturation for 10 min, followed by 40 cycles of 95 °C 15s and 56 °C 1 min. EBV-DNA copy number below 100 copies/ml was considered as negative.

### Evaluation of efficacy

The treatment efficacy was evaluated according to the 2009 RECIST (version l.1) at 3-4 weeks after induction chemotherapy and 1 month after radiotherapy. 1.5 T MRI and CT were performed and measurable lesions of the nasopharynx and neck lymph node metastasis was respectively assessed. Efficacy was defined as progressive disease (PD), stable disease (SD), partial remission (PR), complete remission (CR), and CR + PR = objective response (OR).

### Statistical analyses

The correlation between each clinical parameter and EBV-DNA positive rate was analyzed by chi-square test and Fisher exact probability test. The plasma EBV DNA copy number was converted to a common logarithm value. Samples below the detection limit were given a value of 0. The difference in EBV DNA copy number among groups was compared by Mann-Whitney U test and Kruskal-Wallis test. The correlation between EBV DNA copy number/decrease rate and GTVnx /GTVnd/GTV/tumor regression rate was analyzed by Spearman correlation analysis independent predictors for no residual nasopharynx tumor or neck lymph node metastasis after radiotherapy were analyzed by stepwise logistic regression analysis. The variables were selected by likelihood ratio test according to the following criteria: a variable with a *P* value lower than 0.05 was selected whereas a variable with a *P* value higher than 0.1 was excluded. The area under the ROC curve was compared using function “*roc.test*” embedded in R language package “*pROC*” (version 3.3.1 Foundation for Statistical Computing, Vienna, Austria) with method proposed by DeLong et al. (1988[2]), all other statistical analyses were performed using SPSS 17.0 (IBM SPSS, Chicago, IL, USA). All tests were bilateral, and *P* <0.05 was considered statistically significant.

## Results

### Correlation between EBV DNA level before induction chemotherapy and clinical characteristics of patients

Among the selected 129 patients, 98 were positive for EBV DNA. The EBV positive rate was significantly correlated with age, N stage, GTVnd and GTV (Table 1(Tab. 1)). The EBV positive rate was gradually decreased with age, and was the highest (84.6 %) in patients under 40 years of age. The EBV positive rate was also gradually increased with later lymph node staging, and reached 100 % in N3 patients. Moreover, the GTVnd in EBV positive group was significantly higher compared with EBV negative group (median volume: 15.31 vs. 1.76 cm^3^). After completion of treatment, the EBV positive rate in patients with residual tumor was significantly higher than that in the other patients (90.9 % vs. 68.2 %).

### Univariate analysis of the predictive value of plasma EBV DNA level and tumor volume before and after induction chemotherapy for radiotherapy

**Figure 1:**
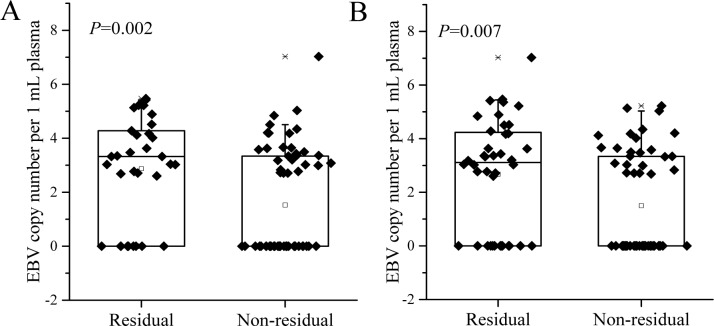
The EBV level in patients with residual tumor after radiotherapy was significantly higher compared with those without residual tumor

In the 98 EBV DNA positive cases, the residual GTVnx and GTVnd after radiotherapy was positively correlated with the EBV DNA copy number after induction chemotherapy (rho = 0.357, *P* <0.001; rho = 0.356, *P* <0.001, respectively), and was negatively correlated with the decrease rate of EBV level before and after induction chemotherapy (rho = -0.298, *P* = 0.003; rho = -0.357, *P* <0.001, respectively). The post-induction chemotherapy EBV level in patients with residual tumor after radiotherapy was significantly higher compared with those without residual tumor (median (range): 3.326 (0.000-5.468) vs. 0.000 (0.000-7.027) for GTVnx; and 3.110 (0.000-7.027) vs. 0.000 (0.000-5.221) for GTVnd, Figure 1(Fig. 1)).

**Figure 2:**
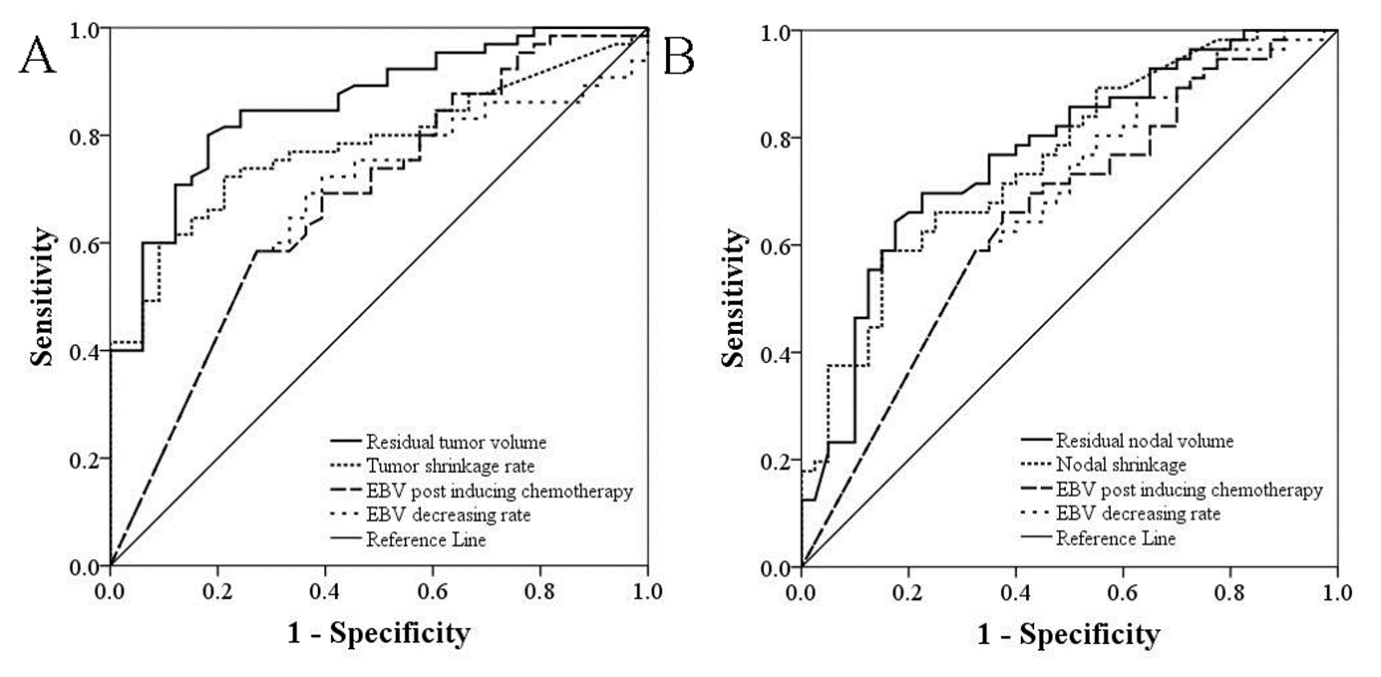
Analysis of sensitivity and specificity by ROC curves

The predictive probability of residual tumor volume after radiotherapy, EBV DNA copy number after induction chemotherapy, as well as tumor regression rate and EBV level decrease rate before and after induction chemotherapy for no residual nasopharynx tumor or neck lymph node metastasis was calculated by univariate logistic regression model. ROC curves were drawn accordingly. As shown in Table 2(Tab. 2) and Figure 2(Fig. 2), the area below ROC curves suggested a higher predictive value of imaging data compared with EBV copy number. For both nasopharyngeal tumors and cervical lymph node metastasis, the area below the ROC curves of residual tumor volume after induction chemotherapy was significantly larger compared with EBV copy number (*P* = 0.0054, and *P* = 0.0309, respectively) and decrease rate (*P* = 0.0017, and *P* = 0.0460, respectively) after induction chemotherapy.

### Multivariate logistic regression analyses of the combined predictive value of tumor volume and EBV copy number for complete remission after radiotherapy

Stepwise multivariate logistic regression analyses were performed with gender, age, TN staging, UICC staging, as well as GTVnx, GTVnd, regression rate, EBV copy number and decrease rate before and after induction chemotherapy as candidate variables. It was shown that T staging, EBV copy number and tumor volume after induction chemotherapy were independent predictors for complete remission after radiotherapy. Nevertheless, the area below the ROC curve drawn based on the probability calculated using the multivariate model (0.851 (95% CI: 0.765-0.936)) was similar to that calculated with tumor volume after induction chemotherapy as the sole predictor (0.851 vs 0.859, z = 0.2841, *P* = 0.776). Therefore, the addition of plasma EBV copy number did not improve the predictive power of imaging data for complete remission after radiotherapy. For cervical lymph node metastases, stepwise logistic regression analyses suggested that lymph volume regression rate was the only independent predictor (Table 3(Tab. 3)).

## Discussion

The identification of a rapid, accurate and sensitive predictor for radiosensitivity is required to achieve precise individualized treatment of NPC. Although tumor burden and EBV DNA level during treatment are the most reliable indicators for the evaluation of short- and long-term efficacy, their predictive value in radiosensitivity has not been previously studied. 

Blood cell-free EBV DNA has been suggested to be closely associated with the apoptosis and proliferation of tumor cells (Choi et al., 2005[1]). In this study, we found that plasma EBV positive rate was correlated with N staging, but not with T staging, suggesting that the EBV DNA level was primarily related to the increased lymph node metastasis instead of primary tumor burden (Sze et al., 2004[11]; Du et al., 2015[3]). Although numerous studies have confirmed the prognostic value of pre-treatment plasma EBV DNA level for long-term survival of NPC patients after treatment (Hou et al., 2011[4]; Peng et al., 2016[9]; Wang et al., 2013[13]; Zhang et al., 2016[15]), it remains a question whether the change in EBV DNA level during induction chemotherapy can be used as a reliable predictor for radiosensitivity. In the 98 EBV DNA positive patients in our study, the residual GTVnx and GTVnd after radiotherapy was positively correlated with the EBV DNA copy number after induction chemotherapy, and was negatively correlated with the decrease rate of EBV level before and after induction chemotherapy, which suggested that the post-treatment EBV level and decrease rate could be used to predict the regression of nasopharynx tumor and neck lymph node metastasis. Further, we analyzed the predictive value of residual tumor volume after radiotherapy, plasma EBV DNA copy number after induction chemotherapy, as well as tumor regression rate and EBV level decrease rate before and after induction chemotherapy for complete remission by univariate logistic regression model. ROC curve analyses revealed that the residual volume after induction chemotherapy has the highest predictive power, followed successively by tumor regression rate, EBV DNA level, and decrease rate after induction chemotherapy. These results suggested that the changes in plasma EBV DNA and tumor volume during treatment could be used to predict the sensitivity of locally advanced NPC patients in response to IMRT. To our best knowledge, the finding has never been previously reported. The current study is mainly limited by the small number of cases. Further studies on a much larger sample size are therefore needed to validate our findings.

## Notes

Yang Song and He Xiao contributed equally as first authors.

## Figures and Tables

**Table 1:**
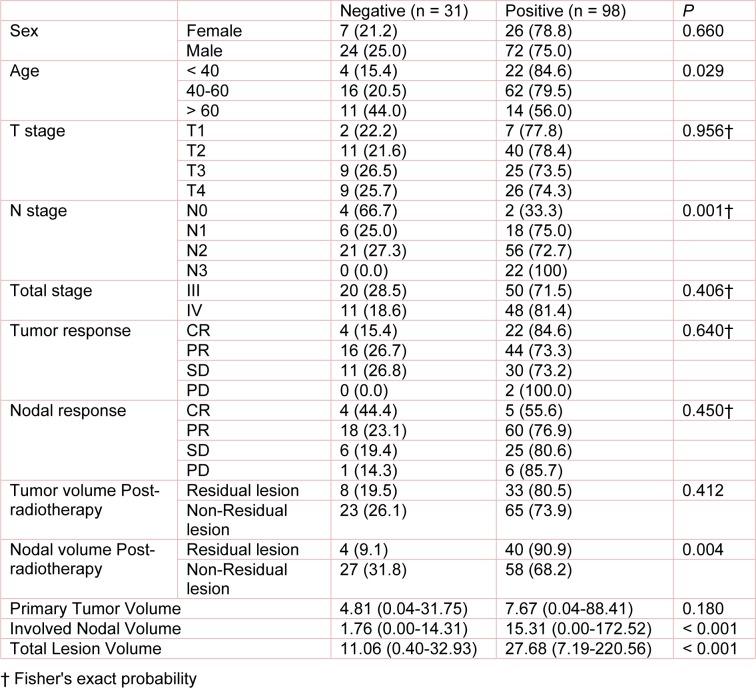
Correlation analyses between clinical and pathological characteristics with EBV infection

**Table 2:**
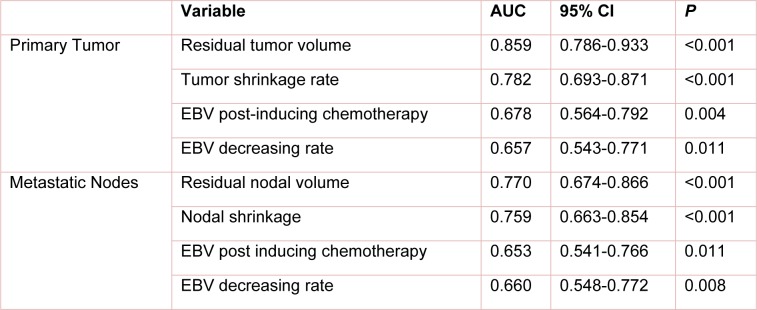
ROC analyses of no residual primary lesion and cervical lymph node metastasis after radiotherapy

**Table 3:**
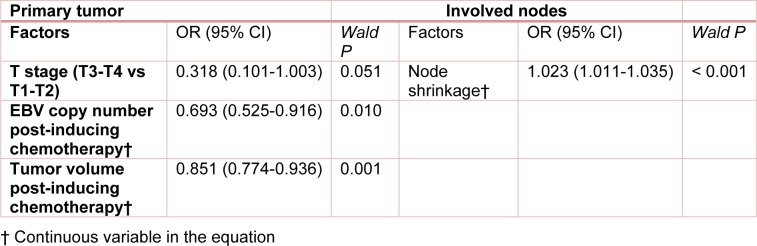
Multivariate logistic regression analyses of predictors for no residual primary lesion and cervical lymph node metastasis after radiotherapy
